# Initial experience of virtual-assisted lung mapping utilizing both indocyanine green and indigo carmine

**DOI:** 10.1007/s11748-020-01565-2

**Published:** 2021-01-03

**Authors:** Masahiro Yanagiya, Yoko Amano, Noriko Hiyama, Jun Matsumoto

**Affiliations:** Department of General Thoracic Surgery, NTT Medical Center Tokyo, 5-9-22 Higashi-Gotanda, Shinagawa-ku, Tokyo, 141-8625 Japan

**Keywords:** Indocyanine green, Thoracic surgery, Virtual-assisted lung mapping

## Abstract

Virtual-assisted lung mapping is a bronchoscopic multiple dye marking technique that facilitates sublobar lung resections for unidentifiable pulmonary tumors. Marking failure reportedly occurs in 10% of cases. To overcome this limitation, we developed indocyanine green virtual-assisted lung mapping that uses indocyanine green in addition to indigo carmine. Here, we report our initial experience of indocyanine green virtual-assisted lung mapping.

## Introduction

Virtual-assisted lung mapping (VAL-MAP) is a preoperative bronchoscopic multi-spot dye marking technique that uses virtual bronchoscopic navigation to localize unidentifiable pulmonary nodules during subsequent surgery [1, 2]. Because of its multiple markings (lung mapping), VAL-MAP facilitates the identification of pulmonary lesions and allows the drawing of resection lines in sublobar lung resections. VAL-MAP is an important technique for thoracic surgeons to achieve sublobar lung resections.

Approximately 10% of VAL-MAP markings are invisible and unidentifiable [1, 2]. Some of the major causes of “marking failure” are anthracosis, pulmonary emphysema, and pleural thickening [1, 2], which are attributed to patient factors. Indigo carmine (IC), a dye used conventionally in VAL-MAP, fades easily and might be inappropriate for these conditions [3].

To overcome the limitations of marking failure because of patient conditions, we used indocyanine green (ICG) dye to visualize the markings more effectively. ICG fluorescence was visualized with a near-infrared thoracoscope. We developed this novel procedure (ICG VAL-MAP) using ICG and IC. Here, we present our initial experience with ICG VAL-MAP.

## Procedure

**Fig. 1:**
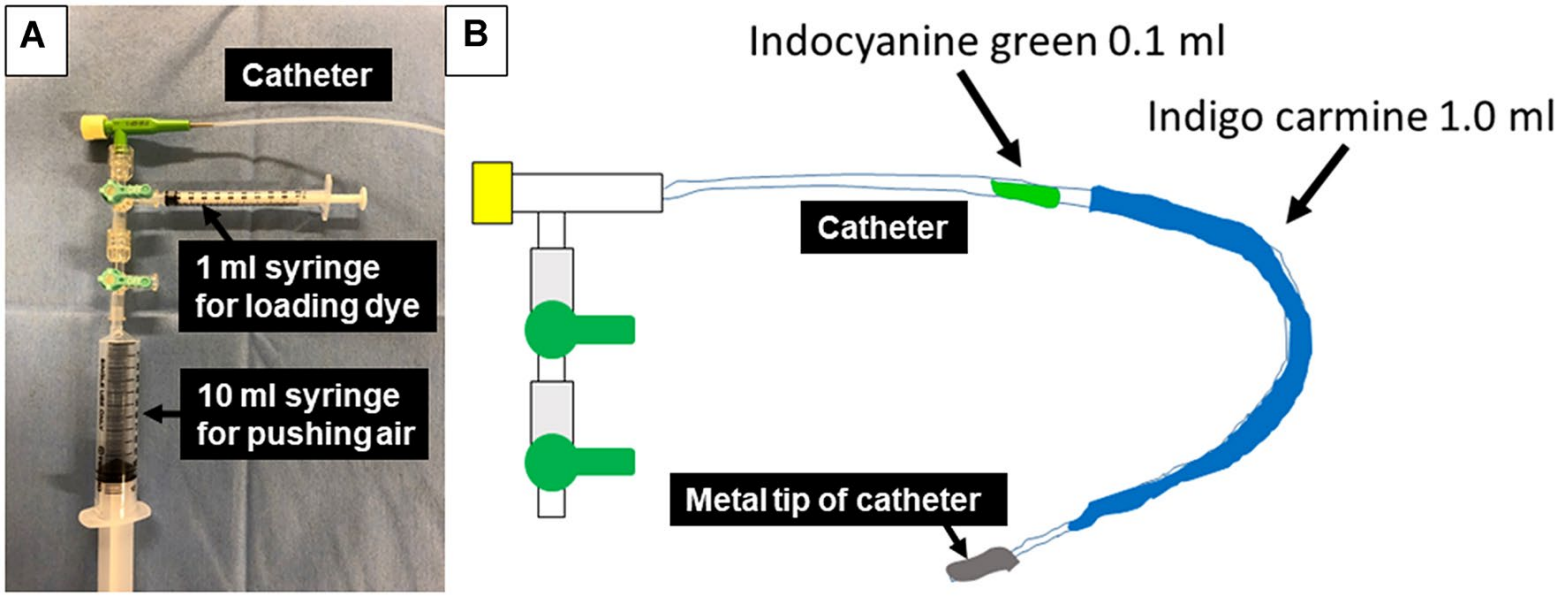
Equipment and preloading of ICG and IC. **a** A metal-tipped catheter was connected by syringes for loading dye and pushing air. **b** Then, 0.1 ml of ICG and 1.0 ml of IC were preloaded into the catheter

**Fig. 2:**
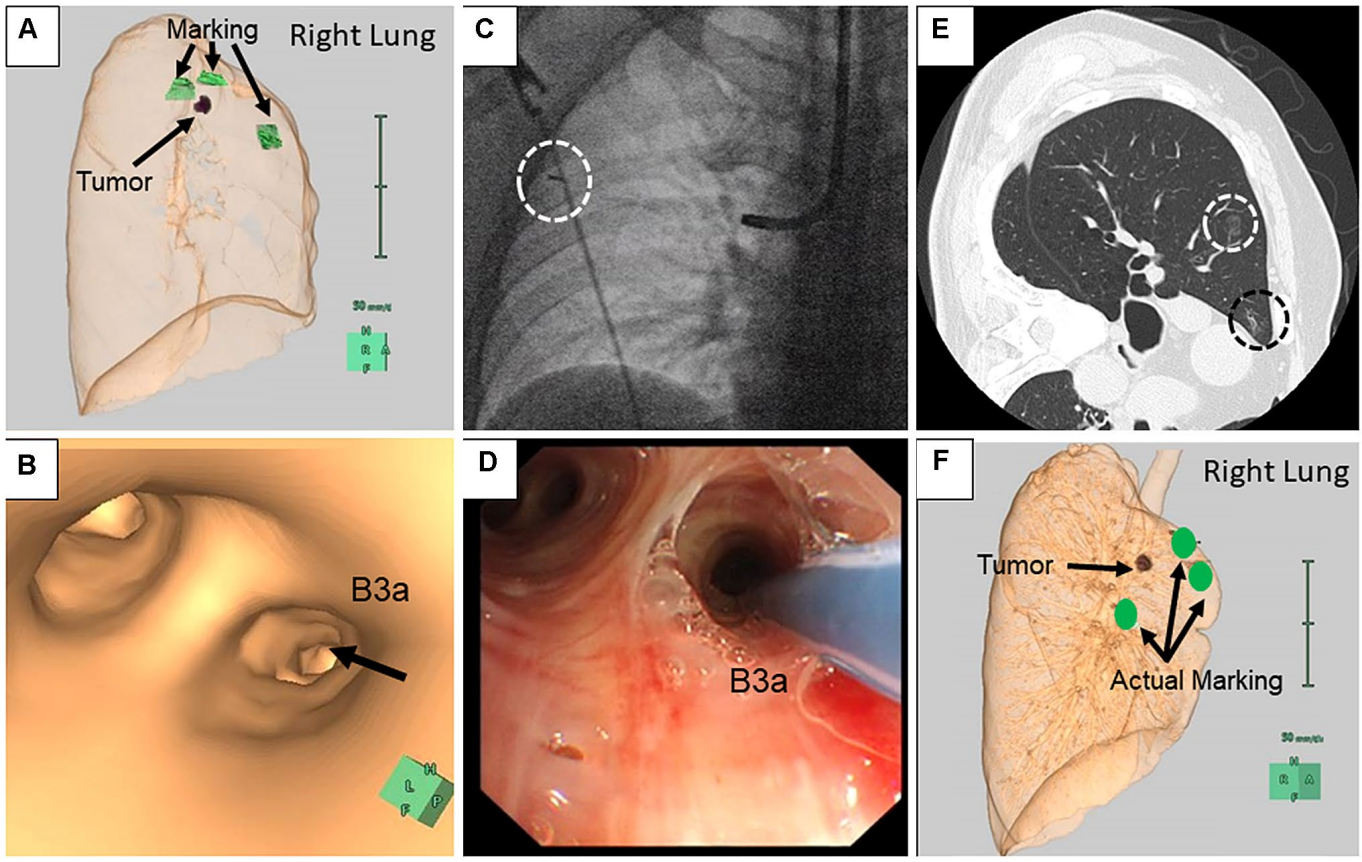
Steps involved in the ICG VAL-MAP procedure. **a**, **b** Before the bronchoscopic procedure, lung mapping was planned and a virtual bronchoscopic navigation image was created using Synapse Vincent. **c** X-ray fluoroscopy confirmed the tip of the catheter (surrounded by a white-dotted circle) reached the pleura. **d** ICG and IC dyes were injected into the targeted bronchus. **e** The post-VAL-MAP chest CT image shows the location of the lesion (surrounded by a white-dotted circle) and the actual marking (surrounded by a black-dotted circle). **f** Three-dimensional image of post-VAL-MAP chest CT was reconstructed for surgery

The mapping procedure, ICG VAL-MAP, was performed similarly to that reported previously [1, 2]. One day before surgery, the patient underwent bronchoscopy for pulmonary markings under local anesthesia and mild sedation. In our novel method, a metal-tipped catheter (PW-6C-1; Olympus, Tokyo, Japan) was initially preloaded with 0.1 ml of ICG and 1.0 ml IC (Fig. 1). Because the mixture of ICG and indigo carmine might cause ICG markings to spread across a wide area, we preloaded ICG and indigo carmine in sequence to avoid mixing them (Fig. 1). Then, the catheter was inserted through the working channel of the bronchoscope into the target bronchus with the aid of a virtual bronchoscopic navigation image (Synapse Vincent; Fujifilm Medical Inc., Tokyo, Japan) (Fig. 2b). Once the tip was confirmed to have reached the pleura under X-ray fluoroscopy, ICG and IC were injected into the target bronchus simultaneously by pushing the plunger of the syringe connected to the catheter, followed by 20 ml of air injection for each mark (Fig. 2c, d). This process was repeatedly conducted for all targeted bronchi. After the bronchoscopic procedure, chest computed tomography (CT) was taken to confirm the actual locations of the markings and the target nodules (Fig. 2e). We created a three-dimensional version of the post-VAL-MAP CT image for the subsequent surgery (Fig. 2f).

During surgery, all markings were assessed by the VISERA ELITE II system (Olympus, Tokyo, Japan) or Da Vinci Xi system (Intuitive Surgical Inc., Tokyo, Japan) equipped with near-infrared fluorescence imaging. We initially evaluated markings based on IC dye marking as conducted in conventional VAL-MAP and then used near-infrared thoracoscopy to evaluate markings dyed with ICG. In the current article, easily identifiable markings were defined as a success, whereas unidentifiable or faint markings were defined as a failure.

## Ethics

Because the bronchial injection of ICG is off-label use in Japan, we conducted ICG VAL-MAP based on ethical approval by the Ethics Committee of NTT Medical Center Tokyo (approval number 19–404) and patients’ written informed consent. This retrospective study used and analyzed the patient data. The present study was approved by the Ethics Committee of NTT Medical Center Tokyo (approval number 20–139).

## Clinical experience

From April 2020 to September 2020, five patients underwent ICG VAL-MAP. The results are summarized in Table 1. All markings and surgeries were performed as planned. Among 20 markings, the number of successful markings by ICG and IC was 19 (95%) and 15 (75%), respectively. All markings were identifiable by ICG or IC. All surgical procedures were wedge pulmonary resections. One patient underwent robotic-assisted thoracic surgery (RATS) wedge resection in addition to RATS lobectomy, which were conducted simultaneously. All lesions were resected successfully with surgical margins more than the diameter of the lesion or 2 cm. There were no adverse events related to the bronchoscopic procedure or use of ICG mixed with IC.

**Table 1:** Characteristics of patients, lesions, markings and surgeries

Patient	Age	Sex	Lesion	Lesion characteristics	Lesion diameter (mm)	Lesion depth (mm)	Number of markings	Bronchoscopic procedure time (min)	Number of visible markings via IC	Number of visible markings via ICG	Surgery	Pathological diagnosis
1	62	M	1	Pure GGN	11	11	4	17	4	3	VATS Wedge	AIS
			2	Pure GGN	5	0					VATS Wedge	Benign change
2	64	M	3	Solid	12	6	5	20	0	5	VATS Wedge	Metastatic tumor
			4	Solid	11	1					VATS Wedge	Metastatic tumor
3	60	M	5	Pure GGN	13	12	3	13	3	3	VATS Wedge	AIS
4	69	M	6	Pure GGN	9	9	5	15	5	5	VATS Wedge	AIS
			7	Pure GGN	5	10					VATS Wedge	AIS
5	76	F	8	Pure GGN	7	5	3	13	3	3	RATS wedge	Benign tumor

*AIS* adenocarcinoma in situ, *GGN* ground-glass nodule, *RATS* robotic-assisted thoracic surgery, *VATS* video-assisted thoracic surgery, *wedge* wedge pulmonary resection

**Fig. 3:**
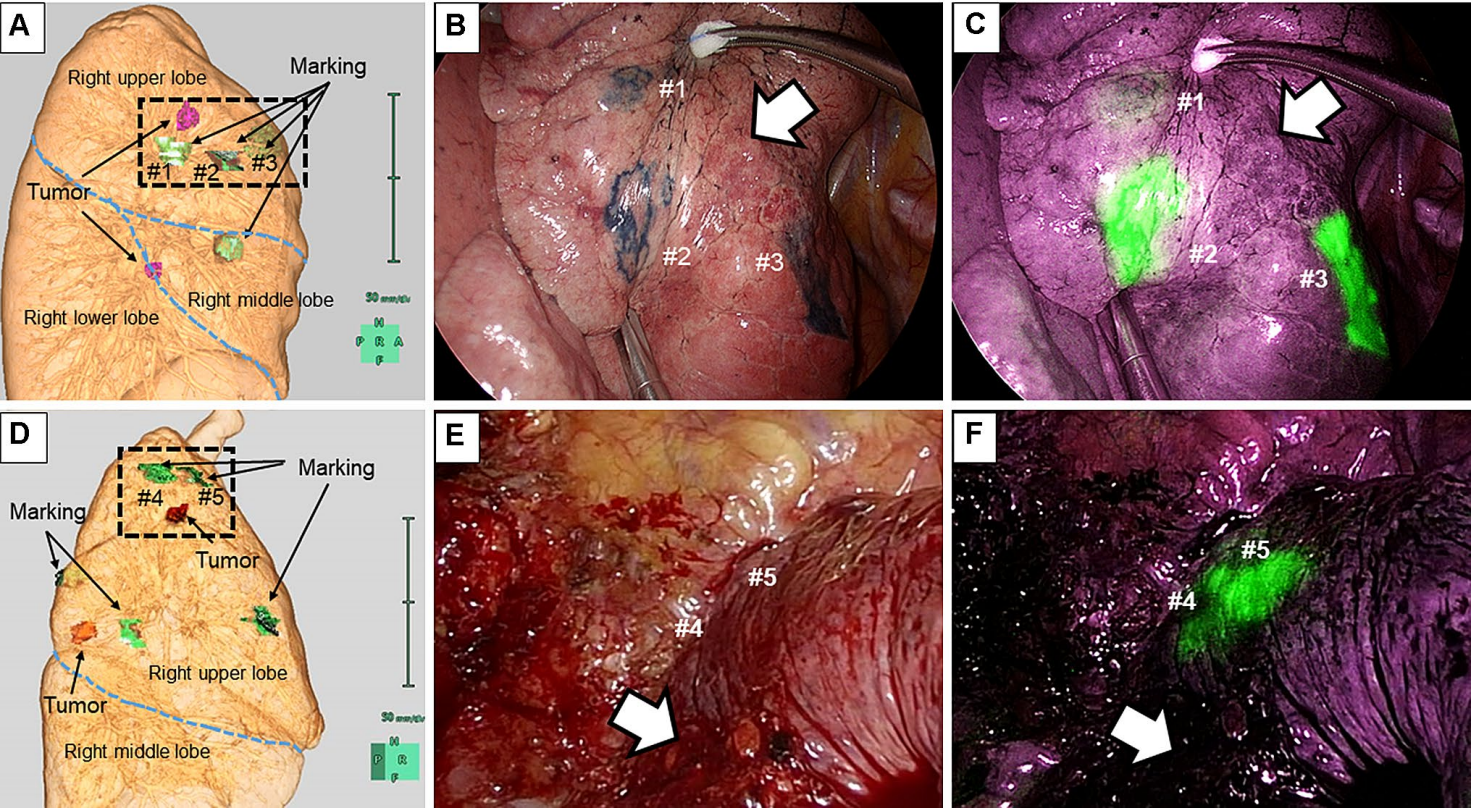
The post-VAL-MAP CT image and actual image of markings. **a**, **b**, **c** The post-VAL-MAP chest CT image and actual intraoperative images of markings in patient #1. IC dye markings are visible (**b**); however, only one ICG marking is faintly visible in marking #1 (**c**). The white arrows indicate the tumor location. **d**, **e**, **f** A post-VAL-MAP chest CT image and actual intraoperative images of markings in patient #2. IC dye markings are invisible due to tight pleural adhesion (**e**). However, the ICG dye markings are visible (**f**). The white arrows indicate the tumor location

Only one marking failure with ICG was noted (patient #1; Fig. 3c). Meanwhile, there were five marking failures with IC (Fig. 3e) in patient #2, who had previously undergone right lower lobectomy through open thoracotomy for pulmonary metastases from colon cancer. Because intraoperative adhesion was extremely tight, all markings were invisible by IC (Fig. 3e). However, ICG fluorescence enabled us to identify all markings and significantly helped the removal of the lesions (Fig. 3f).

## Discussion

We report our initial experience with ICG VAL-MAP utilizing ICG and IC. Our approach has two important aspects. The first one was the resistance to marking failure. As shown in patients #1 and #2, even if IC or ICG marking becomes faint or invisible, the other dye is still visible and can be used to overcome the marking failure. Our initial experience showed that ICG dye marking was effective even under tight pleural adhesion. Although intraoperative adhesion was reported to increase resection failure even with the aid of conventional VAL-MAP [4], our new method may overcome the limitation of the conventional method. In addition, ICG marking might be resistant to marking failure related to central injection or dispersion of the dye from the pleura because ICG and an infrared scope were reported to successfully visualize deep tissues [5]. Second, there was no need for the use of a contrast agent. Previous reports of VAL-MAP using ICG [6] utilized a contrast agent to confirm the location of the actual markings observed in post-VAL-MAP chest CT [5]. Unlike their method, our ICG markings were identifiable by post-VAL-MAP chest CT without the use of a contrast agent (Fig. 2e), which will be useful for patients allergic to contrast agents.

Our method might be improved further. For example, although we preloaded ICG and IC in sequence in the current series, a mixture of both dyes might provide better results.

In conclusion, ICG VAL-MAP using ICG and IC is a promising technique to overcome marking failure. We should accumulate more experience to establish the efficacy and safety of this method.

## Abbreviations

CT: Computed-tomography
IC: Indigo carmine
ICG: Indocyanine green
VAL-MAP: Virtual-assisted lung mapping
